# Construct validity and internal consistency reliability of the Loewenstein occupational therapy cognitive assessment (LOTCA)

**DOI:** 10.1186/s12888-018-1776-x

**Published:** 2018-06-11

**Authors:** Fidaa Almomani, Tamara Avi-Itzhak, Naor Demeter, Naomi Josman, Murad O. Al-momani

**Affiliations:** 1 Department of Rehabilitation Sciences, Faculty of Applied Medical Sciences, Jordan University of Science and Technology, Irbid, 22110 Jordan; 2 Department of Occupational Therapy, York College, The City University of New York, 94-20 Guy R. Brewer BLVD, Jamaica, New York 11451 USA; 3 Department of Occupational Therapy, Joint program, Faculty of Social Welfare & Health Sciences, University of Haifa and Technion, Haifa, Israel; 4 School of Medicine, King Saud University, Riyadh, Saudi Arabia

**Keywords:** Cognitive evaluation, Cognitive performance, Loewenstein occupational therapy cognitive assessment (LOTCA)

## Abstract

**Background:**

Cognitive abilities are essential for children’s development and independence. Various cognitive assessments, standardized in Western cultures, have yet to be investigated for their multicultural suitability.

**Aims:**

To explore the suitability of the Loewenstein Occupational Therapy Cognitive Assessment (LOTCA) for a Jordanian population.

**Methods:**

Observed cases of 442 Jordanian children aged 6–12 were used to perform exploratory factor analyses using principal components with Varimax rotation (construct validity evidence) and to compute Cronbach’s α coefficient (internal consistency reliability).

**Results:**

High total performance on four subscales and a slightly lower total performance on two subscales were observed. Observed performance increased with age on three subtests, whereas a more modest increase was observed on the other three subscales. The expected one-factorial solution confirming the LOTCA’s subscales homogeneity (unidimensionality) structure was found on five of six subscales. Variance explained by the subscales ranged from 39 to 82% and internal consistency reliability measured by Cronbach’s alpha ranged from .42 to .78.

**Conclusions:**

Satisfactory construct validity and internal consistency reliability were demonstrated on two subscales applicable to Jordanian children without adaptation. With adequate cross-cultural adaptation, increasing internal consistency reliability in other subscales could make the LOTCA an effective tool for assessing cognitive abilities in this population.

## Background

Cognitive abilities are skills that form the basis for every human action and enable the performance of tasks ranging from the simple to the complex. Such abilities, including perceptual discrimination, selective attention, memory, and language, are modified by the context in which they developed [1] and represent an evolving product of the interaction between people, activities, and various environmental factors, such as culture and socio-demographic status [2]. These factors determine the type of handling, care, and training given to children, the opportunities for interaction with the environment, and the objects on which they focus [1, 3, 4].

Children vary in their underlying cognitive abilities to process and use information, and these differences affect a child’s learning and academic performance [2]. Indeed, the critical importance of the early childhood years, for cognitive growth and later success, is becoming increasingly evident. Not only are some cognitive abilities, such as reading and mathematical skills, strengthened in school, but cognitive performance may also predict school readiness and scholastic success [3, 4]. Therefore, the assessment of children’s cognitive performance components may provide a basic understanding of their abilities and disabilities in school performance [1]. Early detection of high-risk and special needs children can enable the implementation of appropriate intervention during early learning years [3–5] and influence other aspects of adult life, including social and vocational life, and all life occupations [6–8].

Furthermore, each culture has its own patterns of practices that affect children’s development, behaviors, skills, and attitudes, as well as cognitive performance [3, 4, 9]. Clinicians and researchers worldwide utilize standardized Western tools with strong psychometric properties for clinical purposes, with or without appropriate cross-cultural adaptations. The use of standardized normative data for the purposes of practice with local populations, and the translation and adaptation of Western instruments for use with populations speaking other languages and from different cultures, has drawn increased attention in health-related research and practice [10–12]. A review of cross-cultural studies demonstrated that differences in performance on standardized developmental assessments may be attributed to cultural factors, and that standardized assessments may not be valid or reliable when used to evaluate individuals from a population different from the one in which the original instrument was standardized [9, 13–15].

Currently**,** there are several standardized assessments and screening instruments used by therapists and researchers in the area of cognitive abilities [4, 16], including the Kaufman Assessment Battery (KAB) for Children [17], the Behavior Rating Inventory of Executive Function (BRIEF) [18], and the Woodcock-Johnson Tests of Cognitive Abilities [19]. Although these tests focus on assessing cognitive abilities, Occupational Therapists (OTs) are not eligible to administer the KAB, whereas the BRIEF is a questionnaire, which focuses solely on Executive Functions. The Loewenstein Occupational Therapy Cognitive Assessment (LOTCA), however, was developed for practice by OTs, and both clinicians and researchers routinely use it as a screening tool. One of its main advantages is its ability to assess a range of intact compared to impaired cognitive abilities, thereby enhancing the designing and planning of therapeutic interventions. In addition, the LOTCA was previously used with children in several studies [20] and specifically with Arabic speaking children [3, 4, 21]. The validity and reliability of the LOTCA was previously examined in studies where LOTCA was compared with other instruments. For example, Jang et al. [31] showed that the LOTCA is a valid measure of cognitive abilities for people with intellectual disabilities. They examined psychometric properties of the LOTCA and found correlations with the Pictorial IQ test [31]. The clinical decision to employ this instrument with Jordanian children was based on its strong psychometric properties, minimal language component (rendering translation and test adaptation easier than other Western standardized tests), as well as its extensive practical use by clinicians. For these reasons, we deemed it reasonable to use the LOTCA in the current study with Jordanian children. To date, there is limited documented information on the appropriateness of these standardized Western tests for children of different cultures, though research has suggested that the psychometric properties of a test may vary in different cultural groups [15].

The present study represents an attempt to fill this void by assessing the internal consistency reliability and construct validity evidence of the LOTCA [22] when used with Jordanian children. The purpose of this study was to explore the suitability of the LOTCA for use with Jordanian children, ages 6 to 12, by estimating the adequacy of the psychometric properties of construct validity and internal consistency reliability when administered to Jordanian children. It was hypothesized that the observed items within each of the test’s six subscales would confirm the LOTCA’s (a) homogeneity (unidimensionality) in terms of item structure (construct validity evidence) and (b) consistency structure of the items (internal consistency reliability). The results are expected to indicate the extent to which the LOTCA is valid for use with Jordanian children or whether differentiated norms are needed to accommodate cultural factors.

## Methods

### Study design

This cross-sectional study employed a retrospective design and used quantitative methodology (hypotheses driven).

### Participants

The participants were 442 typically developing (TD) children, aged 6-12 years, enrolled in either public or private schools. Recruitment was conducted via random distribution of 540 flyers to schoolchildren the day before data collection and testing. The flyers contained a description of the study, requested information about the child (e.g., date of birth, known disorders or disabilities), and included a parental consent form. Children with known developmental, learning, or neurological/sensory disabilities, as reported by the teacher and/or the parents, were excluded from the study. Of the 540 flyers, only 98 (18%) recipients refused or were ineligible to participate.

Approval for conducting this study was obtained from the Institutional Research Board (IRB) at King Abdullah University Hospital and the Deanship of Scientific Research at Jordan University of Science and Technology in Jordan. Written parental consent and approval from the educational authorities were received prior to carrying out the study. After receiving the signed parental consent, trained research assistants assessed the cognitive abilities of the subjects using the LOTCA. The research assistants (rehabilitation therapists) were trained by the principle investigator on how to collect data, as well as how to administer and score the LOTCA battery. In order to obtain reliability between reviewers, both the principal investigator and the research assistants assessed and scored the same group of 20 children (10 males and 10 females aged 6-12 years) until 98% compatibility of the results was achieved among them. All subjects were assessed in a quiet environment in the school and parents were not present during the testing (which was administered between 8 am - 10 am or 11 am - 1 pm (i.e., before or after school recess). Average testing time ranged between 45 and 90 min (*M* = 60, *SD* = 12.4).

### Instrumentation

#### Overview of the LOTCA

The LOTCA is a cognitive battery that was developed and designed for use by OTs working in neurological rehabilitation settings. The LOTCA consists of 26 items in six subscales and additional scores for test administration time, attention and concentration. The subscale items ranged from a four- or five- point scale. The individual items and their composite scoring are as follows: 1) *orientation*, includes two items: orientation to place and to time (composite scores range from 2 to 16); 2) *visual perception*, includes four items: object identification, shape identification, overlapping figures and object constancy (composite scores range from 4 to 16); 3) *spatial perception*, includes three items: directions on client’s body, spatial relations, and spatial relations in pictures (composite scores range from 3 to 12); 4) *motor praxis*, includes three items: motor imitation, utilization of objects, and symbolic actions (composite scores range from 3 to 12); 5) *visuo-motor organization*, which examines perceptual-motor integration with spatial components, including seven items: copying geometric forms, reproduction of two- and three-dimensional models, pegboard construction, colored and plain block designs, reproduction of a puzzle, and drawing a clock (composite scores range from 7 to 28); 6) *thinking operations*, which examines categorization and sequencing, including seven items: pictorial classification, object classification (structured and unstructured), pictorial sequencing, and geometrical sequencing (composite scores range from 7 to 35). Results are presented as a profile along all subscales.

The test has an inter-rater reliability coefficient ranging from .82 to .97 for its six subscales. Internal consistency and construct validity were confirmed by Katz, Itzkovich, Averbuch & Elazar (1989) and Najenson et al. (1984) for perception, visuomotor organization and thinking operation [23, 24]. In their study on people with stroke and brain injury, Katz et al. (1989) found the LOTCA to have discriminant validity for all subscales aside from the identification of objects [23].

Although the LOTCA was standardized in Western populations and has not yet been standardized for use in other populations, it has the potential for evaluating individuals from non-Western populations [20]. As mentioned above, several studies have used the LOTCA to study the cognitive abilities of Arab children [3, 4, 21]. The results of these studies suggest that culture may explain the differences in performance and scores between cultural groups and the lack of cultural adaptation may have affected the construct validity and internal consistency reliability of the LOTCA. However, these studies were performed without prior testing as to whether the LOTCA’s normative data required cross-cultural adaptations.

A composite score for each of the LOTCA’s six subscales was calculated by totaling the scores of the relevant items. The maximum score on the test is 123, and the minimum score is 27. A higher score indicates better cognitive performance. Raw scores were used in this study, as the LOTCA battery has not yet been standardized for Jordanian children.

The English version of LOTCA, including the instruction manual, was translated into Arabic using a backward-forward translation process [21]. Discrepancies in the translation of specific items were discussed until a consensus was reached. A pilot study with 20 children was carried out in order to examine the readability of the initial versions of the translated scale; the items were revised accordingly and then were piloted to 20 other children. At that point, researchers unanimously agreed that no more amendments were needed. This translated Arabic version of the instrument was then retranslated into English by a bilingual speaker who was unfamiliar with the original versions of the instrument. This process of backward translation was evaluated by ten bilingual professors working at Arabic universities.

The scores of the instrument in evaluating the translation, ranged from 0 (not similar) to 1 (similar) and a cut off score of at least 0.80 was identified in order to assess the adequacy of the Arabic translated version, implying that 80% or more of the evaluators agreed that the backward-translated item had the same meaning as the original item. A score below 0.80 suggested a possible problem with the translation. After the translation stage was completed and modifications were made, all translated items achieved the cutoff score of 0.80.

#### Data analysis and management

Descriptive statistics (*M* and *SD*) were computed to describe the distributions of the test’s six subscales and socio-demographic data. Exploratory factor analyses (EFA) using principal components extraction model with Varimax rotation were performed on the six subscales.

The *Kaiser’s criterion,* used to determine the number of factors to retain, suggests retaining all factors that are above the eigenvalue of 1 [25, 26]. All 26 items were found to have enough variance and were maintained in the factor analysis and Cronbach’s alpha computations. The minimum Eigenvalue for rotated factors was established at one [27] and the criterion for determining whether an item loaded substantially on a subscale was established at .50 [27]. Cronbach’s alpha was computed for the items within each of the six subscales. Cronbach’s alpha [28] indicated 0.7 to be a satisfactory reliability coefficient; however, lower thresholds are occasionally used in the literature. There are no widely accepted criteria for defining a strong vs. moderate association. Portney and Watkins (2015) suggest values of *r* ranging from .50 to .75 to represent moderate to good relationship and those ranging from .75 and above to be good to excellent relationship [29].

EFA generated an expected one-factor solution showing the expected unidimensionality factor structure for five of the six LOTCA subscales. The EFA yielded a two-factor solution showing multidimensionality for the subscale *Thinking Operation.* An EFA without Pictorial B (the cross loading item) generated a one-factor solution with a slightly increased amount of explained variance and Cronbach’s α, confirming the expected homogeneity (unidimensionality) structure of this LOTCA subscale and was used in the analysis. Significance was set at the 0.05 probability level. All operations were carried out using SPSS Version 20 (SPSS, 2012).

## Results

The 442 Typically Developing (TD) children who participated in this study ranged in age from 6 to 12 years, with a mean of 9.12 ± 1.9 years. The sample was almost equally divided between males and females (51.4% males), urban vs. rural areas (48.6% urban), and public vs. private schools (49% in public schools). The majority (96.2%) of the subjects lived with both parents and 46.4% of the parents had an average yearly family income of JD 6000 ($ 8600).

Associations between cognitive functioning and independent demographic variables (i.e.; child’s age, area of residence, hours of sleep, parent’s occupation etc.) were analyzed using a multivariate linear regression model. This description is provided in our previous publication [21].

The total observed mean performance score of four of the six domains is located toward the higher end of the possible composite range score, indicating high observed performance on these domains [*orientation [M* = 13.32 (*SD* = 3.32)- composite scores ranging from 2 to 16; *visual perception*, *M* = 14.53 (*SD* = 1.36)- composite scores ranging from 4 to 16; *spatial perception*, *M* = 11.30 (*SD* = 1.33) - composite scores ranging from 3 to 12; and *motor praxis*, *M* = 11.42 (*SD* = .99), composite scores ranging from 3 to 12]. While the total mean score of the other two subscales is slightly lower, performance on these subscales is still quite high: [*visuo-motor organization*, *M* = 22.35 (*SD* = 4.23) - composite scores ranging from 7 to 28; *thinking operations*, *M* = 24.71 (*SD* = 4.36- composite scores ranging from 7 to 31] (see Table 1).

**Table 1:** Means and Standard Deviations of the LOTCA Six subscales for the Seven Age Groups (*N* = 442)

Age groups (Years)	Number	*Orientation*	*Visual Perception*	*Spatial Perception*	*Motor Praxis*	*Visuo-Motor organization*	*Thinking operations*
		Possible Range 2-16 *Mean SD*	Possible Range 16-4 *Mean SD*	Possible Range 3-12 *Mean SD*	Possible Range 3-12 *Mean SD*	Possible Range 7–28 *Mean SD*	Possible Range 7–31 *Mean* SD
6	46	10.98 (3.42)	13.48 (1.64)	10.50 1.83)	11.11 (1.10)	17.63 (4.48)	20.04 (4.55)
7	61	11.80 (3.74)	13.79 (1.36)	10.74 (1.78)	11.23 (1.17)	20.34 (3.04)	21.74 (4.17)
8	65	13.46 (2.59)	14.58 (1.11)	11.48 (1.03)	11.32 (.970)	22.17 (4.25)	24.98 (3.92)
9	75	14.07 (3.08)	14.59 (1.28)	11.55 (.90)	11.48 (.89)	22.80 (3.88)	25.09 (3.79)
10	70	13.44 (3.62)	14.69 (1.19)	11.50 (.88)	11.63 (.74)	22.79 (3.89)	25.24 (3.60)
11	69	14.62 (2.66)	15.00 (1.26)	11.51 (1.34)	11.55 (.94)	24.20 3.27)	26.58 (3.29)
12.	56	14.71 (2.42)	15.27 (.92)	11.52 (1.16)	11.52 (.94)	25.18 (2.68)	27.98 (2.68)
Total	442	13.41 (3.32)	14.53 (1.36)	11.30 (1.33)	11.42 (.99)	22.35 (4.23)	24.71 (4.38)

*Note.* LOTCA (Loewenstein Occupational Therapy Cognitive Assessment (Itzkovich et al., 2000)

As shown in Table 2, the observed mean performances on each of the six LOTCA subscales were significantly correlated with one another. Of the 15 Pearson correlation coefficients, there were 12 significant small correlations (ranging from *r* = .22; *p* ≤ .001 to *r* = .44; *p* ≤ .001) and three moderate to good coefficients ranging from (*r =* .53; *p* ≤ .001 to *r* =. 69; *p* ≤ .001)*.* These correlations suggest that all subscales measure the same construct and provide satisfactory levels and evidence of construct validity.

**Table 2:** Pearson Zero Order Correlation Coefficients for Correlations between the LOTCA Subscales (*N* = 442)

Domain	*1.*	*2.*	*3.*	*4.*	*5.*	*6.*
Orientation	–					
Visual Perception	.33*	–				
Spatial Perception	.40*	.31*	–			
Motor Praxis	.23*	.31*	.43*	–		
Visuo- Motor Organization	.44*	.53*	.38*	.35*	–	
Thinking Operations	.42*	.57*	.43*	.35*	.69*	–

*Note.* LOTCA (Loewenstein Occupational Therapy Cognitive Assessment (Itzkovich et al., 2000)

^*^p ≤ .001

Results of the exploratory factor analysis (EFA) and Cronbach’s α for the subscale *orientation* generated the expected one-factor solution with an eigenvalue of 1.64, confirming the LOTCA’s homogeneity (unidimensionality) factor structure and thus providing construct validity evidence. Accounting for approximately 82% of the variance, with a Cronbach’s α of .76, results show a satisfactory level of internal consistency reliability. Therefore, this subscale may be used with Jordanian children ages 6 to 12 without cross-cultural adaptation (see Table 3). Results for the *visomotor organization* subscale yielded an expected one-factor solution with an eigenvalue of 3.06, confirming the LOTCA’s homogeneity (unidimensionality) factor structure and providing adequate construct validity evidence. Accounting for approximately 44% of the variance with a Cronbach’s α .78 this subscale exhibits a satisfactory level of internal consistency reliability. Consequently, this subscale may be used with Jordanian children ages 6 to 12, without cross-cultural adaptation (see Table 3).

**Table 3:** LOTCA Subscales (Not Requiring Cultural Adaptation) Factor Loadings, Eigenvalues, Communalities, Percent of Explained Variance and Cronbach’s Alpha (*N* = 442)

Domain/Measure	Loading	Initial Eigenvalue	Communalities	% of Variance explained	Cronbach’s α
*Orientation*					
1.Orientation to Place	**.910**	1.64	.820	82.08	.76
2. Orientation to time	**.910**		.820		
*Visuo-Motor Organization*					
13.Copying geometric forms	**.712**	3.06	.507	43.81	.78
14. Two dimensional model	.476		.226		
15. Pegboard construction	**.696**		.485		
16. Colored block design	**.637**		.405		
17. Plain block design	**.712**		.507		
18. Reproduction of a puzzle	**.717**		.514		
19. Drawing a clock	**.649**		.422		

*Note.* LOTCA Loewenstein Occupational Therapy Cognitive Assessment (Itzkovich et al., 2000)

Boldface type indicates primary loading for each measure

Eigenvalue = pre-rotation column sums of squared loadings

^a^Communalities = calculated per item, based on a one-factor solution

^b^Cronbach’s α reported for primary loadings (boldface type)

EFA for the subscale *visual perception* yielded an expected one-factor solution with an eigenvalue of 1.58, confirming the LOTCA’s homogeneity (unidimensionality) factor structure and providing an adequate level of construct validity accounting for approximately 40% of the variance. The Cronbach’s α of .47 does not provide a satisfactory level of internal consistency reliability. This subscale may not be used with Jordanian children ages 6 to 12 without adequate cross-cultural adaptation to increase its internal consistency reliability (see Table 4).

**Table 4:** LOTCA Subscales (Requiring Cultural Adaptation) Factor Loadings, Eigenvalues, Communalities, Percent of Explained Variance and Cronbach’s Alpha (*N* = 442)

Domain/Measure	Loading	Initial Eigenvalue	Communalities	of Variance explained	Cronbach’s α
*Visual Perception*					
3.Object identification	**.478**	1.58	.229	39.57	.47
4. Shape identification	**.617**		.381		
5.Overlaping figures	**.726**		.526		
6. Object constancy	**.669**		.447		
*Spatial Perception*					
7. Directions on Ca Body	**.726**	1.75	.527	58.34	.63
8. Spatial relations	**.820**		.673		
9. Spatial relations on pictures	**.742**		.551		
*Motor Praxis*					
10.Motor imitation	**.640**	1.54	.410	51.60	.47
11. Utilization of objects	**.767**		.589		
12. Symbolic actions	**.741**		.550		
*Thinking Operation*		2.645		44.09	.74
14.Categorization	**.693**		.480		
*15. ROC unstructured*	**.772**		.598		
*14. ROC structured*	**.736**		.542		
*15. Pictorial sequence A*	**.638**		.400		
*16. Geometric sequence*	**.600**		.360		
*17. Logic questions*	**.510**		.260		

*Note.* LOTCA Loewenstein Occupational Therapy Cognitive Assessment (Itzkovitch et al. 2000). Boldface type indicates primary loading for each measure

Eigenvalue = pre-rotation column sums of squared loadings

Communalities = calculated per item, based on a one-factor solution

Cronbach’s α reported for primary loadings (boldface type)

EFA for the *spatial perception* subscale generated an expected one-factor solution with an eigenvalue of 1.75, confirming the LOTCA’s homogeneity (unidimensionality) factor structure for this subscale, demonstrating a satisfactory level of construct validity evidence. This solution accounted for approximately 58% of the variance. Cronbach’s α was .63, indicating a questionable level of internal consistency reliability. Consequently, this subscale may not be used with Jordanian children ages 6 to 12 without cross-cultural adaptation to increase its internal consistency reliability (see Table 4).

EFA for the *motor praxis* subscale generated an expected one-factor solution with an eigenvalue of 1.54, confirming the LOTCA’s homogeneity (unidimensionality) factor structure demonstrating a satisfactory level of construct validity. This solution accounted for approximately 52% of the variance. Cronbach’s α was .47, exhibiting an unacceptable level of internal consistency reliability evidence. Consequently, this subscale may be not be used with Jordanian children ages 6 to 12 without cross-cultural adaptation to increase its internal consistency reliability evidence (see Table 4).

Factor analysis for the modified *thinking organization* subscale generated a one-factor solution exhibiting the expected unidimensionality with and eigenvalue of 2.645. The LOTCA’s homogeneity (unidimensionality) factor structure of this modified subscale was confirmed, demonstrating unacceptable level of construct validity. This solution accounted for approximately 44% of the total variance. Cronbach’s α was .74 exhibiting a satisfactory level of internal consistency reliability. Although construct validity and internal consistency reliability evidence were established, this modified subscale may not be used with Jordanian children ages 6 to 12 without a significant cross-cultural adaptation designed to further study the possible consequences of eliminating the item Pictorial B from the subscale (see Table 4).

## Discussion

The purpose of the present study was to explore the suitability of the LOTCA for use with Jordanian children. The findings show that overall, the translation and use of the LOTCA with Jordanian children ages 6 to 12 demonstrated sufficient additional cross-cultural adaptation efforts, the LOTCA could be an effective “culture-free” tool for assessing the cognitive abilities of Arabic speaking Jordanian children ages 6 to 12.

### Construct validity - LOTCA internal subscale structure

The computation of Pearson correlations provided additional validation for estimating the construct evidence of the LOTCA’s test structure. The six LOTCA subscales are presumed to assess different aspects of cognitive ability and as such, are expected to be positively related to one another. Indeed, the observed correlations among the six LOTCA subscales were all significant. While a small-observed association was found between *Motor Praxis* and *Orientation,* the rest of the correlations represented mostly moderate associations. Therefore, while supporting the test structure proposed by the LOTCA between the subscales, the mostly modest correlations also suggest that each subscale item represents a distinct construct of cognitive ability contained in the cognition and visual perception functions inherent in the developmental theory being tested in the LOTCA. Thus, in order for the LOTCA to maintain its effectiveness in assessing cognitive abilities in this population, all six subscales should be retained, though additional cross–cultural research is needed for four of the six subscales.

### Construct validity – LOTCA within subscales Unidimensionality structure

The EFA generated five one-factor solutions and one two-factor solution, confirming the expected homogeneity (unidimensionality) factor structure of the items within five of the six LOTCA subscales. All solutions had eigenvalues that satisfied the *Kaiser’s* criterion used to determine the number of retaining factors*.* The initial EFA for the *thinking operations* subscale yielded a two-factor solution and the homogeneity (unidimensionality) factor structure of the LOTCA subscales was not confirmed.

The *thinking operations* subscale involves higher mental processes such as executive function, problem solving, abstraction, and logical operation [22] and socio-cultural background is known to affect such thinking patterns [30]. Therefore, this subscale may be sensitive in identifying differences between children from different cultures. This claim is supported by Jang, Chern & Lin (2009) who showed that the LOTCA is a valid measure of cognitive abilities among people with intellectual disabilities. In examining known group validity, *thinking operations* was the most sensitive subscale for identifying differences between the ID and the healthy groups [31].

The item Pictorial Sequence B was loaded above the cutoff point on both factors, becoming a cross loading item [26, 32]. A possible explanation is that each factor defines a distinct cluster of interrelated items, and items in cross**-**loading situations are usually eliminated from the analysis [26, 32]. In the present study, Pictorial Sequencing B can be eliminated, as the skills measured by this item are measured by the other items in the subscale.

The pictorial sequence has various underlying components, such as cognitive abilities of judgment, sequence, organization, and comprehension, which may already be measured by other items in the thinking operations subscale. A cross-cultural adaptation process can help assess whether the subscale contains enough information to adequately cover what it is intended to measure without this specific item.

In the present study, the criterion for determining whether an item loaded substantially on a subscale was established at .50 [27]. In twenty-four of the 26 items contained in the LOTCA four of the six subscales, namely, *Orientation, Spatial Perception, Motor Praxis* and *Thinking Operations* subscales loaded above the established cutoff point, ranging from .61 to .91. It is important to note that the loadings of these items demonstrated a satisfactory construct validity evidence for these subscales [26]. The relatively high loadings of these items provide evidence of the strength of the correlations between them and of the meaningful contribution of each item within each of the four subscales [33].

The loadings of the item *Object Identification* (.47), part of the *Visual Perception* subscale and *Two Dimensional Model* (.48), part of the *Visomotor Organization* subscale, were slightly below the cut-off point. These relatively low loadings show that their contribution to each subscale is less meaningful [33]. These findings also indicate that the dimensions of the *Visual Perception* and *Visu-Motor Organization* subscales are not sufficiently accounted for by their items and thus do not demonstrate satisfactory evidence for construct validity for these subscales [26]. *Object Identification* and *Two Dimensional Model* are simple items, originally prepared for people with brain injuries. Thus, it is not surprising that TD children completed these items easily and obtained high scores on them, creating a ceiling effect.

These findings affect the ability of Arabic speaking Jordanian clinicians to interpret the composite score of these subscales as a reflection of all the subscale items [34]. Thus, for the LOTCA to be an effective tool for assessing cognitive abilities with Jordanian children there is a need for cultural adaptation in order to achieve satisfactory levels of construct validity.

### Internal consistency reliability – LOTCA subscales

In the present study, the estimates of internal consistency reliability were obtained using Cronbach’s alpha. As the coefficient alpha is affected by test length and subject to inflation, it has been suggested that alpha should be calculated for each of the subscales rather than for the entire test [28, 35, 36]. Therefore, in this study, estimates of internal consistency validation were calculated for each of the six subscales individually. The calculated coefficients generated demonstrated different levels of internal reliability evidence among the six subscales. Satisfactory levels of internal consistency reliability evidence (α = .76) were found for *Orientation, Visomotor* and T*hinking operations* subscales. For example, *Visomotor* and *Thinking operations* were significantly correlated at the .69 level. Hence, the skills needed to perform the *Visomotor* subscale are also the basis of the *Thinking operation* subscale and indicate a satisfactory degree to which the items comprising each subscale measure the same construct [34] in the present study, regardless of culture. For example, although the item ‘pegboard construction’ is under *Visomotor organization*, its underlying components include a categorization component, such as which color the subject should choose. Such item homogeneity directly affects the ability of a Jordanian clinician to interpret the LOTCA composite clinical score as a reflection of all the measures comprising each of these subscales. The items on these subscales are correlated with each other regardless of culture differences.

A questionable level of internal consistency reliability evidence (α = .63) was found for the subscale *Spatial Perception*, indicating an insufficient ability to measure the same construct. In a previous study (Josman et al., 2010) which compared the cognitive functioning of Israeli and Palestinian children using the LOTCA, the *spatial perception* subscale was included in different factors in each group. In the Israeli group, *spatial perception* was included in a factor named Visuo-motor organization and sequence abilities and loaded .62 while in the Palestinian group it was included in a factor named *temporal and spatial perception* and loaded .45. The study authors noted that these findings were due to cultural differences such as age of entry to the educational system and engagement in different cognitive tasks.

Unacceptable levels of internal consistency reliability evidence (α = .47) were found for the *Visual Perception* and *Motor Praxis* subscales, clearly indicating that the measures comprising these subscales do not jointly measure the same construct and lack item homogeneity. Since the subscale’s measures are linearly combined into a composite score, the concern for item homogeneity directly affects the ability of clinicians to interpret composite scores on these subscales. Since the evidence of measures of homogeneity was absent, these subscales require cultural adaptation before they can be of utility to Arabic speaking Jordanian clinicians.

The value of Cronbach’s alpha for the *Orientation, Visu-Motor* and T*hinking* subscales was within satisfactory level of internal reliability (76), indicating a relatively low level of error variance for the measures contained in each of these subscales; thus they can be considered reliable as a single construct even without cultural adaptation. The Cronbach’s alpha for the other three subscales, *Spatial Perception, Visual Perception* and *Motor Praxis,* was below the accepted level of internal reliability, indicating a high level of error variance for the measures contained in these subscales to be considered as a single construct. This might be due to the high dimensionality in the measures, more often associated with low values for Cronbach’s alpha while not necessarily indicating that the measures are not adequate or reliable [37]. The extent to which the measures in each subscale exhibit high dimensionality should be further researched, and the high level of error variance in the measures needs to be addressed. The high fraction of error variance associated with the measures contained in these three subscales limits their clinical utility and thus the ability of a clinician to perform a reliable assessment.

### Limitations

Although there are several important implications for the current study, some limitations should be addressed. First, future psychometric research on the LOTCA should include other aspects of validity and reliability not undertaken in the present study. There is also a need to assess the association between test performance and academic achievement. Second, children with known disabilities were excluded from the current study. While including those children might have added to the discriminating value of the LOTCA, it is recommended to address this issue in future studies. Third, the administration and especially scoring of the LOTCA requires some training of personnel, because not all items are non-verbal. Therefore, some items are not suitable for people with hearing/speech problems. In addition, the test is time-consuming. Therefore, it might be that some aspects of the results are attributable to instrument administration.

## Conclusions

The translation and use of the LOTCA demonstrated satisfactory evidence of construct validity and internal consistency reliability in two subscales, *Orientation* and *Visu-motor organization*. The one-factor solutions obtained for each subscale confirmed the LOTCA subscale homogeneity (unidimensionality) structure and the above-satisfactory levels of Cronbach’s alpha attested to the consistency of the measures within these subscales and hence, to satisfactory internal consistency reliability evidence. These findings suggest that these two subscales were found to be free of cultural bias and could be used with Jordanian children without the need for cultural adaptation. With adequate cross-cultural adaptation efforts to adapt the other four subscales, the LOTCA may be used as an effective tool for assessing cognitive abilities with Jordanian children ages 6 to 12. Future research should include additional representative samples. Future psychometric research on the LOTCA to include other aspects of validity and reliability not undertaken in the present study is also needed, as well as assessing the association of test performance with academic achievements.

## Abbreviations

BRIEF: Behavior Rating Inventory of Executive Function
EFA: Exploratory Factor Analyses
KAB: Kaufman Assessment Battery
LOTCA: The Loewenstein Occupational Therapy Cognitive Assessment
TD: Typically developing
